# Crystal Ball – 2015

**DOI:** 10.1111/1751-7915.12269

**Published:** 2015-01-28

In the following articles, leading researchers in the field of microbial biotechnology speculate on the technical and conceptual developments that will drive innovative research and open new vistas over the next few years.

